# Latest trends in bioimaging and building a proactive network of early-career young scientists around bioimaging in Europe

**DOI:** 10.1242/bio.059630

**Published:** 2022-12-21

**Authors:** Hana Valenta, Nathan Quiblier, Valerio Laghi, Clément Cabriel, Justine Riti

**Affiliations:** ^1^ Department of Chemistry, Lab for Nanobiology, KU Leuven, Leuven 3001, Belgium; ^2^ GDR Imabio, CNRS, Villeurbanne 69100, France; ^3^ Inria, AIstroSight Team, Villeurbanne 69100, France; ^4^ LIRIS UMR5205, University of Lyon, Villeurbanne, Paris 75015, France; ^5^ Institut Pasteur, CNRS UMR 3738, Paris, Paris 75005, France; ^6^ Institut Langevin, ESPCI, Paris, Gif-sur-Yvette 91190, France; ^7^ Laboratoire d'Etudes et de Recherches en Immunoanalyse (LERI), CEA, Gif-sur-Yvette, France

**Keywords:** Fluorescence microscopy, Bioimaging, Label-free microscopy

## Abstract

Biological research is in constant need of new methodological developments to assess organization and functions at various scales ranging from whole organisms to interactions between proteins. One of the main ways to evidence and quantify biological phenomena is imaging. Fluorescence microscopy and label-free microscopy are in particular highly active fields of research due to their compatibility with living samples as well as their versatility.

The Imabio Young Scientists Network (YSN) is a group of young scientists (PhD students, postdocs and engineers) who are excited about bioimaging and aim to create a proactive network of researchers with the same interest. YSN is endorsed by the bioimaging network GDR Imabio in France, where the initiative was started in 2019. Since then, we aim to organize the Imabio YSN conference every year to expand the network to other European countries, establish new collaborations and ignite new scientific ideas.

From 6-8 July 2022, the YSN including researchers from the domains of life sciences, chemistry, physics and computational sciences met at the Third Imabio YSN Conference 2022 in Lyon to discuss the latest bioimaging technologies and biological discoveries. In this Meeting Review, we describe the essence of the scientific debates, highlight remarkable talks, and focus on the Career Development session, which is unique to the YSN conference, providing a career perspective to young scientists and help to answer all their questions at this career stage. This conference was a truly interdisciplinary reunion of scientists who are eager to push the frontiers of bioimaging in order to understand the complexity of biological systems.

## Teaser

**Summary:** This Meeting Review highlights the latest trends in bioimaging discussed at the Third Imabio YSN Conference 2022 in Lyon which was organized by and dedicated to early-career young scientists.

## INTRODUCTION

### Why do we need bioimaging?

Bioimaging relies on a series of techniques developed to visualize biological organization and activity, ideally non-invasively and in real time. It aims to interfere as little as possible with life processes to preserve the native functions and structures. Bioimaging spans the observation of subcellular structures at the nanoscale level over entire cells, tissues up to entire multicellular organisms.

Fluorescence microscopy together with label-free microscopy are key techniques of bioimaging, allowing us to visualize cellular and biochemical events at the molecular level under physiological conditions, which leads to better understanding of how an entire organism works. Nowadays, these microscopy methods cover a whole range of research domains, from the development of genetically encoded fluorescent proteins and nanosensors, through advanced microscopy techniques (e.g. super-resolution microscopy), to the image analysis. The knowledge acquired thanks to bioimaging can then open a way to a better drug design and development, advances in biomedical imaging, personalized medical therapies and improvements in the general well-being in society.

Today, bioimaging is becoming increasingly popular in many research laboratories, either to answer biological questions or to improve the current imaging technologies and bring innovations. In France, the research group GDR Imabio, which consists of 140 laboratories and core facilities, brings together scientists from interdisciplinary domains, who seek to push the frontiers of knowledge of various biological topics through the development of new strategies in microscopy. In 2019, a spin-off of the GDR Imabio, the Imabio Young Scientist Network (YSN) was created, with the goal of creating a group focused on young scientists, PhD students, postdocs and engineers, who are at the beginning of their careers and interested in bioimaging, and aims to create a proactive network of researchers with the same interest. Since 2019, the Imabio YSN organizing committee (Fig. 3A) have organized a conference every year to expand the network, establish new collaborations and ignite new ideas around bioimaging.

**Fig. 1.**
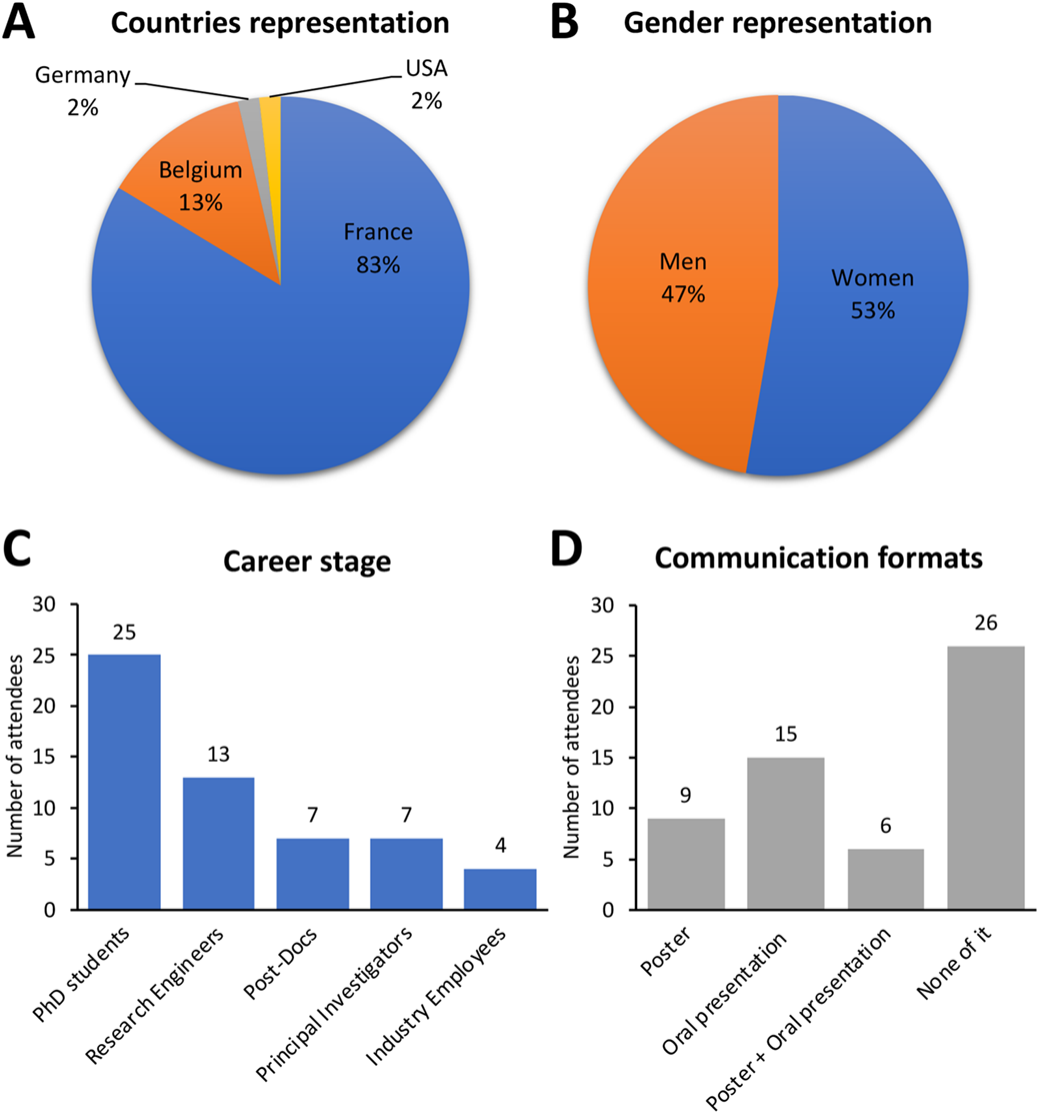
**Participant distribution at the Third Imabio YSN conference.** The graphs compare the location of their research lab (A), their gender (B), their career stage (C) and the format of the communication they presented (D).

The Third Imabio YSN Conference 2022 took place in Lyon 6-8 July 2022 with around 60 participants present on site. Most of the participants were from France, but there were also participants from Belgium, Germany and the US (Fig. 1A). The gender distribution was very balanced (Fig. 1B).

The format of the Imabio YSN Conference is slightly different from most other scientific conferences. First of all, it is truly focused on the young scientists, which is also reflected in the statistics - the largest group of attendees at the YSN conference 2022 was the one of PhD students, followed by research engineers and postdocs (Fig. 1C). The general goal is to give them as much space as possible to present their work and research in various communication formats (Fig. 1D) and help to build their network. Throughout the entire YSN conference, the young scientists are the main speakers, and their presentations represent the bulk of the program. For the same reason, there is only one keynote lecture at the end of each day, which is given by an invited senior researcher. The organization committee is also entirely composed of young scientists. Additionally, part of the Imabio YSN conference is always dedicated to a Career Development session to help the young scientist to grasp the principles and dynamics of the job market in different spheres of academia and industry and to provide them with an overview of their future career options. Finally, the conference is organized to be environmentally friendly with a minimum waste production (see ‘Green conference’ below).

## What's new in bioimaging: highlights

The scientific program of our Third Imabio YSN conference was divided into three different sessions. Within the session dedicated to the Bioinformatics and Optics, Clément Cabriel (ESPCI, Paris, France) talked about high density single molecule localization using event-based sensors, which is an alternative new approach of acquiring and processing single-molecule localization microscopy (SMLM) data based on an event-based (or neuromorphic vision) sensor (Cabriel et al., 2022 preprint). His talk was followed by a presentation from Jiachuan Bai (Institut Pasteur, Paris, France), on ShareLoc – an open platform for sharing localization microscopy data (Ouyang et al., 2022). From the afternoon part of this session, we would like to highlight the talk of Ko Sugawara (ENS, Lyon, France) who presented his recently developed method called ELEPHANT (Sugawara et al., 2022), which enables tracking of cell lineages with a modest investment of time and effort compared to the existing methods (Fig. 3C). This session was closed by a lecture given by Charles Kervrann (INRIA, Rennes, France) who talked about statistical and artificial intelligence methods for live-cell fluorescence imaging.

Franziska Bierbuesse (KU Leuven, Belgium) presented, in the session about Photochemistry and Labeling, a method called photochromism-enabled absolute quantification (PEAQ) that provides an absolute measurement of the analyte concentration or activity in cells using photochromic single-fluorophore biosensors (Bierbuesse et al., 2022). Corentin Rousset (Université Lyon 1, Lyon, France) with his talk, ‘New tools for multicolor dSTORM imaging’ gave insights into the new method based on blinking particle standards and conditions, which enables long-lived imaging over several weeks (Provost et al., 2019)*.* Direct stochastic optical reconstruction microscopy (dSTORM) is one of the super-resolution microscopy techniques that allows researchers to overcome the diffraction limit of light and acquire images with very high resolutions. Hana Valenta (KU Leuven, Belgium) presented a method of separation of spectrally overlapping fluorophores using intra-exposure excitation modulation (Valenta et al., 2021), which gives new possibilities for multiplex imaging in complex samples. At the end of the session, Thomas Chaigne (Fresnel Institute, Marseille, France; Fig. 3D) gave a keynote lecture about research concerning photoacoustic imaging: towards functional, single-cell resolution imaging a couple millimeters deep.

In the session of the last day, which was called ‘Biology: answers to biological questions thanks to the fluorescence imaging’*,* we heard about various biological themes approached by different fluorescence microscopy strategies. For example, Franceline Juillard (Institut NeuroMyoGène, Lyon, France) presented her results from deciphering the three-dimensional structure of latent herpes simplex virus 1 genomes by lattice-SIM^2^, which is a type of super-resolution microscopy improved to be able to perform structured illumination microscopy (SIM) with a doubled resolution (SIM^2^) compared to the conventional SIM (Neil et al., 1997). This technique allows researchers to discriminate the finest sub-organelle structures even smaller than 100 nm. Yad Ghavi-Helm (ENS, Lyon, France) gave the final lecture in this session presenting the latest news concerning regulating gene expression in 3D during embryonic development.

Among the many high-quality oral presentations, we would like to put in the spotlight that of Laura Breimann (Harvard Medical School, Boston, MA, USA), who uses fluorescence microscopy to study mechanisms of transcription repression by an X-specific condensin in *Caenorhabditis elegans* (Breimann et al., 2022). Laura won the Oral Presentation Contest and was awarded a prize sponsored by BioAxial.

At the end of the first day of the conference, we organized a classic poster session (Fig. 3B), which gave rise to many passionate scientific debates and also in general helped to break the ice among participants.

## Career Development session

The Career Development session is a unique feature of the Imabio YSN Conference. Many of the young scientists (at least in France), such as PhD students, know only the pathway within the academia and they rarely have much other professional experience. Most students trained in the university system have only academic training. They often have many questions about their future and about the options they have, but do not have access to more senior contacts who could answer these questions. It also appears that many students lack information about academic careers and related positions and selection processes. The organizing committee of Imabio YSN conferences is also composed of PhD students, postdocs and young engineers in the same or very similar situations. We understand the need for bridging these information gaps for young researchers to be made aware of hiring processes, salaries and job content. Therefore, we decided to implement Career Development session to every Imabio YSN Conference. We are convinced that this session, in the form of round tables and imaging platform visits, enables face-to-face communication between the early-stage career scientists and the industry and start-up representatives.

In the 2022 edition, we decided to arrange three sessions of round tables focused on ‘Academia’, ‘Industry’ and ‘Start-up creation’. For the Academia round tables, we welcomed our keynote speaker Thomas Chaigne, then Adrien Bosseboeuf (postdoc at ENS, Lyon, France) and Jacques Brocard (head of the PLATIM imaging platform, ENS, Lyon, France). Discussion topics included researcher position applications, research funding, work and personal life balance, team management, teaching and other types of responsibilities. The round tables on Industry were in presence of Paul Barthélémy (Abbelight, Cachan, France), Jonathan Reboulet (Lipics-Services, Lyon, France), Alexandra Schroeder and François Stransky (both BioAxial by Telight, Paris, France). The meeting tackled questions such as advertising academic skills in applications, job content and responsibilities, management and the differences between development in industry and academic research. Finally, for the round tables focused on the ‘Start-up creation’, participants discussed with Julien Chartron (ALPAO, Lyon, France), Jens Hasserodt (Molsid, Lyon, France), and Arthur Villesuzanne (Inria Startup Studio, Lyon, France). They shared their feelings about entrepreneurship, and how it is different from an academic career or a classic industrial career, both professionally and personally, in terms of motivation and scientific production. Moreover, a huge part of their talk was dedicated to explaining how they found help when they decided to start their companies. Particularly Arthur Villesuzanne is working in the type of structure (Inria Startup Studio) that supports project owners to start creating a digital Deeptech startup, which made the discussion very fruitful.

In the end, the debates within round tables were very dynamic, and many participants found the testimonies very honest and appreciated being able to hear about the advantages and disadvantages of all of these spheres from the people who actually work in them.

During the Career Development session, participants could also choose to visit an imaging platform of ENS Lyon, PLATIM. They were split into small groups and with the guidance of the permanent staff they could acquire hands-on knowledge on various microscopes’ setups. For example, they could experiment with a two-photon microscope (Fig. 3E) or with an atomic force microscope (Fig. 3F).

It is important for the early-career stage scientists to have meetings such as the third Imabio YSN conference that are focused on them and that allow them to discuss their projects without any boundaries. The resulting interactions will help them to build their network, which will be useful for future collaborations. Moreover, the Career Development session is an essential part of this conference, bringing to light various career possibilities after finishing the PhD and answering necessary questions from the young scientists.

We plan to keep organizing the Imabio YSN conference as we see it as an incubator for big future scientific minds and leaders. We intend to expand the existing format and invite young scientists from all over Europe to create a solid YSN network with stable structure and regular events. At the same time, we aim to raise awareness of the amount of pollutants that are produced by science-related travel and organization. As we want to enlarge our YSN community over Europe, it will certainly require an effort. However, a big change is nothing less than the sum of individual contributions and we strongly believe that everyone can change their travel habits, not only while attending a conference. We will encourage participants of the Fourth Imabio YSN conference, which is planned for summer 2023, to act responsibly and think about the ‘greener’ options.

## Green conference

Attending a conference in person has many advantages over an online conference, such as direct social interaction and easier communication. Nevertheless, scientists physically travelling to the conference site will increase their carbon footprint and the event itself can generate a lot of food, plastic and water waste (We need greener conferences, 2019). We care about our planet, and we aimed to make the Third Imabio YSN conference environmentally friendly.

**Fig. 2.**
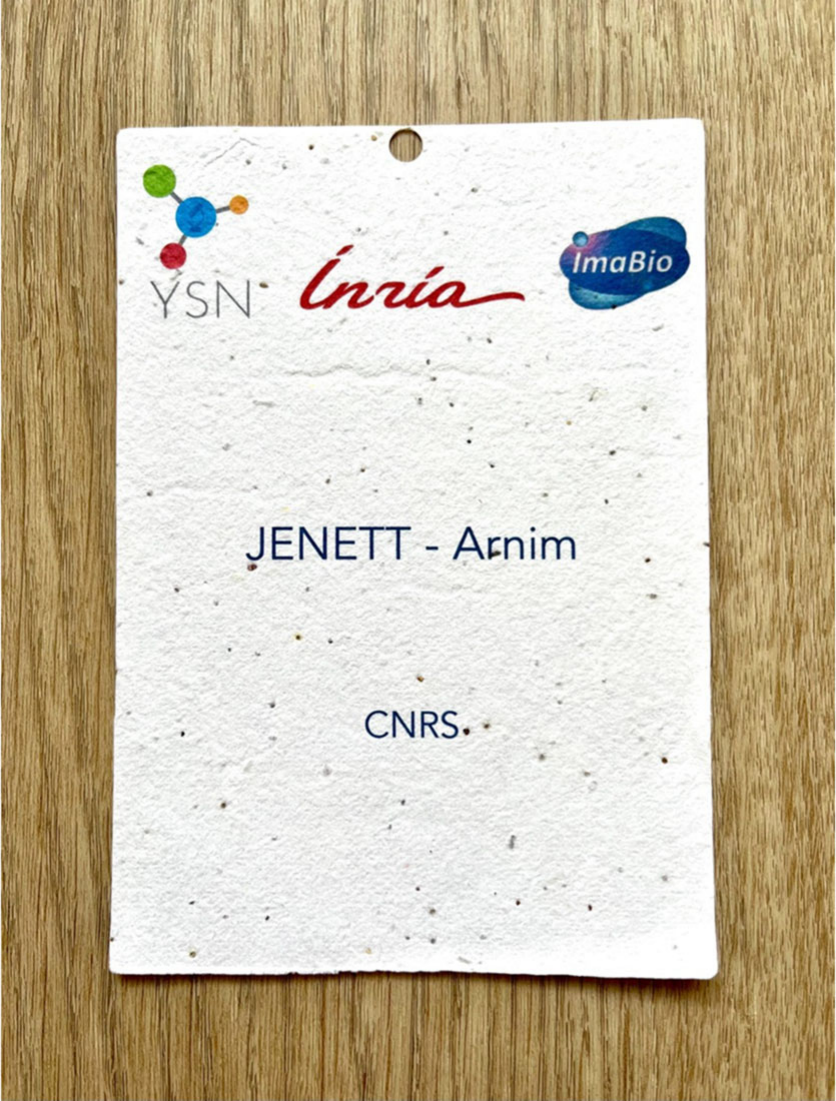
Ecological badge of one of the participants.

For this reason, participants were encouraged to travel by train rather than by airplane or by car, and in the end truly no one came by air transport. All participants also brought their own mugs for coffee breaks and a strictly minimal number of paper cups was purchased. We provided badges (Fig. 2) for everyone made from recycled seed paper, which do not need to be thrown into the waste containers after use but planted in the ground to help grow new plants.

**Fig. 3.**
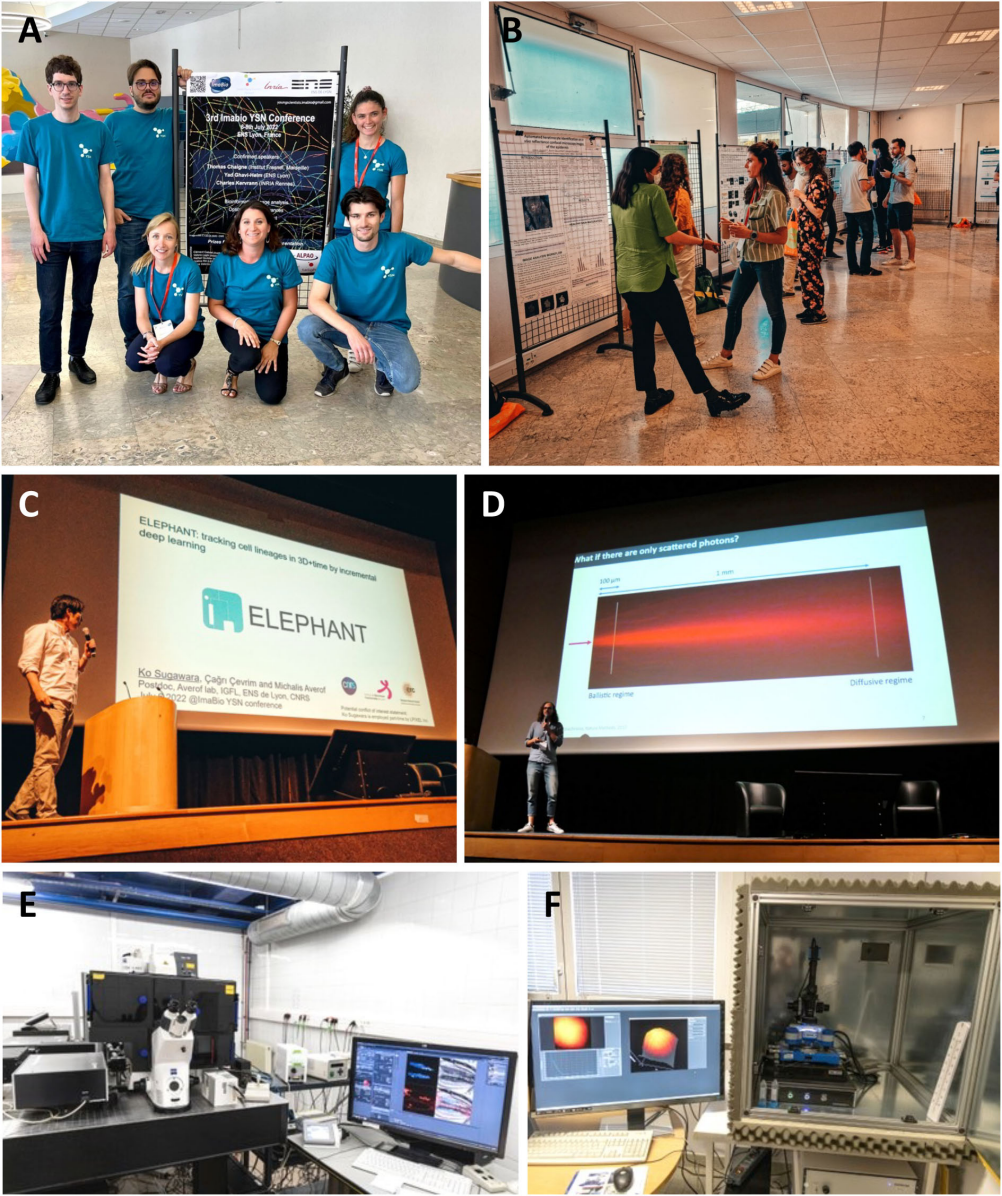
**(A) Organizing committee of the Third Imabio YSN Conference 2022: from the top left: Clément Cabriel, Valerio Laghi, Justine Riti, Hana Valenta, Laetitia Lécot-Gauthé and Nathan Quiblier.** (B) Discussions at the poster session. (C) Invited speaker Thomas Chaigne giving a lecture about photoacoustic imaging. (D) Ko Sugawara giving a short talk about his ELEPHANT image analysis method. (E) Zeiss LSM 710 two-photon microscope. (F) Bruker Nanowizard ULTRA Speed Atomic Force Microscope, both microscopes are at the PLATIM imaging platform of ENS Lyon.

Indeed, the conferences with physical attendance are more appreciated by most of the scientists, but it is our responsibility to decide how ‘green’ or wasteful we organize them. Here, we would like to appeal for a smart behavior not only from organizers but also from the participants.

Questions that we could ask ourselves are, for example:

- How can we limit the food waste? Is there a zero-waste association nearby, which could help?
- Can we actively recycle the waste generated during the conference? How?
- How many participants will come, and by which means of transport?
- As a participant, do I need to travel all around the world to give a 30 min talk? Is it worth it?
